# Descriptive analysis of post-stroke patients in a neurological physical therapy unit

**DOI:** 10.3389/fneur.2023.1056415

**Published:** 2023-02-28

**Authors:** Mercedes Paniagua-Monrobel, Isabel Escobio-Prieto, Eleonora Magni, Alejandro Galan-Mercant, David Lucena-Anton, Elena Pinero-Pinto, Carlos Luque-Moreno

**Affiliations:** ^1^Department of Physiotherapy, Faculty of Nursing, Physiotherapy and Podiatry, University of Seville, Seville, Spain; ^2^Neurological Physiotherapy Unit, Virgen del Rocio University Hospital, Seville, Spain; ^3^Institute of Biomedicine of Seville (IBIS), Seville, Spain; ^4^Department of Nursing and Physiotherapy, University of Cádiz, Cádiz, Spain; ^5^MOVE-IT Research Group, Department of Physical Education, Faculty of Education, Sciences University of Cádiz, Cádiz, Spain; ^6^Biomedical Research and Innovation Institute of Cádiz (INiBICA) Research Unit, Puerta del Mar University Hospital, University of Cádiz, Cádiz, Spain; ^7^Intell-SOK (TIC-256) Research Group, Department of Informatics Engineering, University of Cadiz, Cádiz, Spain

**Keywords:** stroke rehabilitation, outpatients, inpatients, neurological rehabilitation, physical therapy department, hospital, early ambulation

## Abstract

**Introduction:**

Physical therapy (PT) is the mainstay treatment in functional recovery after suffering a stroke. It is important in the acute phase of hospitalization after a stroke and later in the ambulatory phase.

**Patients and methods:**

The present study aimed to analyze the data provided by the clinical history (CH) of people with stroke (pwS) who received PT treatment in order to establish a “preferential patient profile” (PPP) that may benefit more from an early PT treatment. This was an observational, descriptive, and cross-sectional study. A total of 137 pwS who had been treated with PT were selected. Information provided age, gender, stroke type and localization, and start and end dates of the different PT treatments. A descriptive analysis of the variables was conducted using absolute frequencies and percentages for the qualitative variables. Student's *t*-test or the Mann–Whitney *U*-test was used to determine the relationship between the time and variables “stroke type,” “outpatient,” and “occupational therapy.” The Kruskal–Wallis *H*-test was applied for the “localization” variable.

**Results:**

Of the entire sample, 57.7% were men, 65% had an ischemic stroke, and 48.9% had a stroke on the left side. The patients with hemorrhagic stroke had an increased number of hospital PT sessions (*p* = 0.01) and were younger (59.58 years) than patients with ischemic stroke (65.90 years) (*p* = 0.04).

**Discussion and conclusion:**

Our results do not show significant differences between the persons < 65 years and the number of outpatient physiotherapy sessions performed, although the resulting values are close to significance. Our results suggest that the PPP is a young person, with a hemorrhagic and left or bilateral stroke.

## Introduction

Cerebrovascular accident (CVA), due to its high rate and prevalence, is a disease of great health and social impact. In Europe, the estimated prevalence of stroke is 9.2%, with a rate of 191.9/100,000 people/year (1). In Spain, according to the Spanish Institute of Statistics, in the year 2020, a total of 25,817 people died of cerebrovascular diseases (2). It is estimated that between 25 and 74% of persons who survive this disease require assistance or become fully dependent on their activities of daily living (3). The main residual post-stroke disabilities include motor disorders, paralysis, cognitive deterioration, dysphagia, and speech disorders (4). Therefore, CVA is one of the pathologies with greater social and economic repercussions at the international level, as well as one of the most important causes of disability in adults (5, 6). Currently, the social impact of this disease is even greater than the increase of CVA cases in young adults of working age, with ~5–10% of strokes occurring in people under 50 years of age (7), which results in the loss of years of healthy life and productivity (8). In the last decades, Spain has been advancing in diagnosing and treating these persons, improving their care and recovery (9).

Depending on the phase of the disease, there are two main scopes of assistance in pwS (9): (1) the hospitalization phase or acute phase (from the onset of the symptoms to the hospital discharge), in which the treatment must be applied by a multidisciplinary team, including physical therapy (PT) (10); and (2) the subacute phase (3–6 months after the stroke), in which the PT treatment is essential to prevent complications and recover the patient's maximum functional capacity possible, to maximize his/her personal autonomy and his/her family and social reintegration (11). In this phase, PT can be performed as outpatient treatment (home care), in a hospital scope (12), and a medium- or long-stay center or hospice, depending on the clinical and/or social situation of the pwS. Hospital PT after suffering a stroke produces improvements in all patients, regardless of their age; however, age reversely predicts a good functional result (13) (the younger the patient, the better the results). There is a third and final phase of sequelae, in which some authors report a functional improvement after 12 months, in cases who received PT treatment, and a progressive functional deterioration in the absence of specific therapies (14, 15).

The role of PT in pwS must begin after an initial evaluation aimed at establishing a PT diagnosis from the results obtained in it. This diagnosis is based on the International Classification of Functioning, Disability, and Health (ICF), which considers deficiencies in bodily functions and structures, activity limitation, participation restriction, and existing contextual factors, both environmental and personal (12, 16). This allows for establishing the prognosis and the treatment objectives and developing a PT intervention plan (17, 18).

The pwS's degree of recovery depends on different factors, such as the amount of brain tissue affected, age, localization of the damaged area, early rehabilitation, and environmental and psychosocial factors (19). Studies such as that of Kleim and Jones (20) support the idea of experience-dependent plasticity, which is understood as the capacity of the brain to re-adapt in response to an experience or task (20). Although the capacity of the brain to adapt and compensate for the effects of an injury is lower in adults than in earlier stages of life, it has been reported that the capacity to recover is present in all ages (21). Moreover, there are genetic and non-genetic protective factors that influence the process of neuronal plasticities, such as age, education, the importance of the injury, and the behavioral characteristics of the patient (6, 22).

Thus, the aim of PT is to help the pwS to maintain the existing abilities after the CVA, recover the lost abilities, and learn new abilities (23) through neuroplasticity.

The aim of the present study was to associate the variables, stroke type (hemorrhagic/ischemic), brain localization, and person's age and sex, with the number of sessions received in the different phases of both hospital and outpatient PT, as well as the waiting time between sessions. Specifically, we analyzed whether the preference criteria for starting the outpatient PT treatment, monitored in the Neurological PT Unit of a Spanish hospital, for pwS under 65 years of age, correlated with significant differences in the number of PT sessions performed, in order to establish a “preferential patient profile” (PPP) that could benefit more from an early start of the outpatient PT treatment.

## Materials and methods

### Study design

A descriptive, cross-sectional, and correlational study was carried out with 137 pwS who had been treated with PT in the hospital phase or ambulatory phase in the Neurological PT room of Virgen del Rocio University Hospital (Seville, Spain).

### Participants

**Figure 1:**
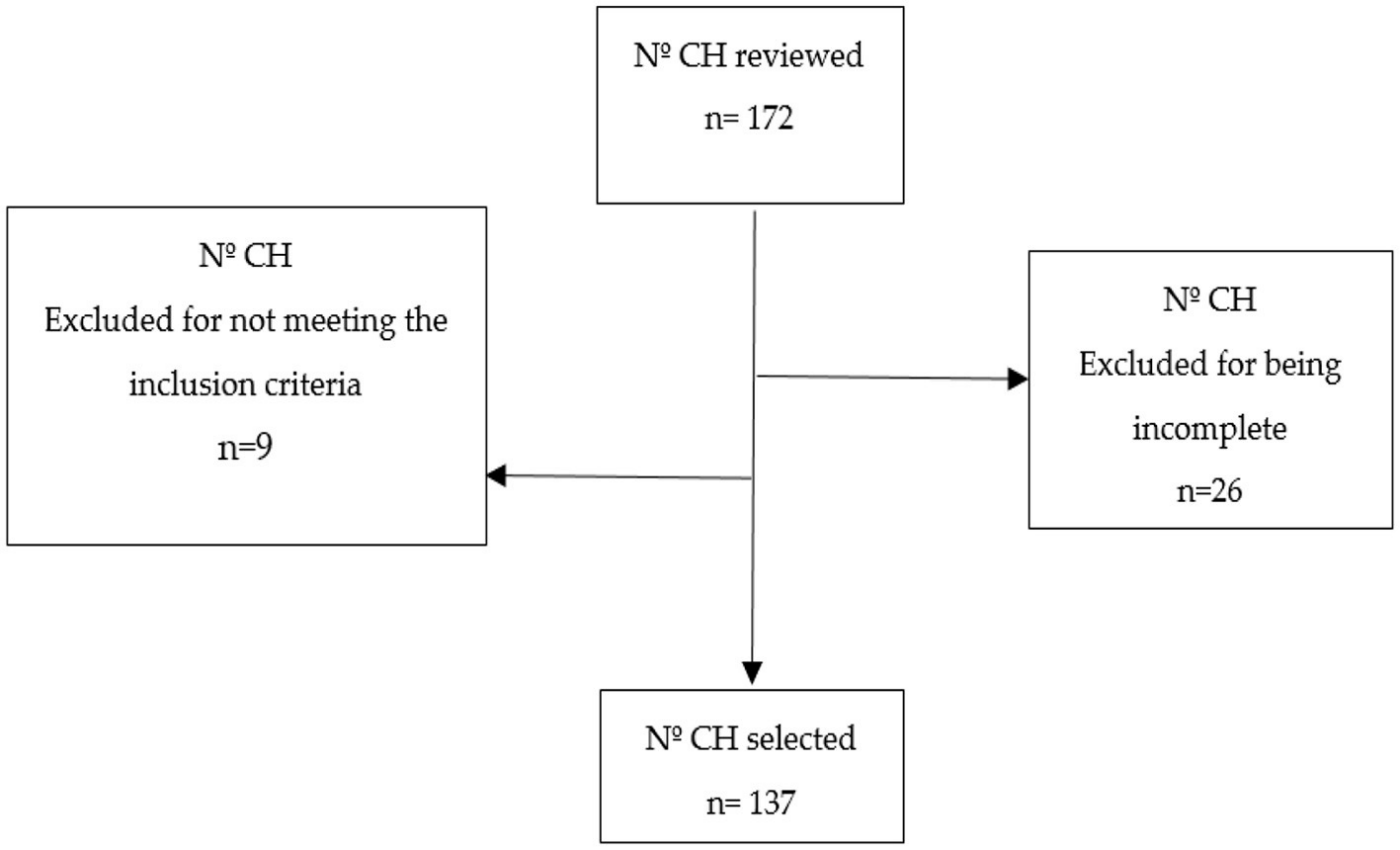
Sample selection process. CH, clinical history.

The inclusion criteria were as follows: pwS treated in the PT unit (hospital or ambulatory phase) affected by ischemic or hemorrhagic stroke during the period between 10 July 2014 and 25 April 2018 (4 years). On the other hand, the study excluded those persons who had suffered more than one stroke event, died during the study, or received the PT treatment in the sequelae phase (at least 1 year after the stroke) (Figure 1).

A total of 172 clinical histories (CHs) were reviewed, of which 35 were discarded for being incomplete or not meeting the inclusion criteria. Finally, 137 pwS were included, who were attended to in the abovementioned period (Figure 1).

### Interventions

After fulfilling the eligibility criteria, the CH of each pwS was analyzed, guaranteeing the safety and confidentiality of the gathered data at all times. The data were extracted from the computer-based registry, which included the following variables:

- Demographic data: age and sex.
- Clinical PT treatment; the start and end dates from the hospital data: stroke type (hemorrhagic/ischemic); lesion side (right/left/bilateral); start date of the hospital PT treatment; hospital discharge date (which coincides with the end of the hospital PT treatment); start date of the outpatient PT treatment (in the neurological PT room); PT discharge date; having or not having received home PT treatment from the rehabilitation and PT mobile units (from the hospital discharge to the start of the outpatient PT treatment); and having or not having received occupational therapy treatment in the outpatient phase.

### Outcome measures

A senior PT with over 10 years of clinical experience in neurological PT collected the CH at the beginning of the analysis. The gathered data were registered in a database created with Microsoft Excel 2013 software for Windows.

### Statistical analysis

The statistical processing of the data was conducted with IBM SPSS statistical package Version 19.0. A descriptive analysis of the study variables was conducted using absolute frequencies and percentages for the qualitative variables. The quantitative variables, based on their asymmetry, were summarized as M ± SD (mean and standard deviation) and range (minimum and maximum) or P50 [P25–P75] (median, interquartile range). The normality of the distributions was verified using the Kolmogorov–Smirnov test. To determine the relationship between the time and variables “stroke type,” “outpatient,” and “occupational therapy,” a Student's *t*-test or Mann–Whitney *U*-test was conducted, depending on the normality of the variables. In the case of the variable “localization,” the Kruskal–Wallis *H*-test was applied since the time did not follow a normal distribution. After verifying the times' distribution normality, the association among them was determined through Spearman's correlation coefficient (rho), as they did not show a normal distribution. The level of statistical significance was established at *p* < 0.05.

### Role of the funding source/ethics

No sponsor was involved in the study design; in the collection, analysis, and interpretation of data; in the writing of the report; and in the decision to submit the article for publication.

The trial design complied with the ethical guidelines set in the Declaration of Helsinki and was approved by the Institutional Research Ethics Committee of Virgen Macarena and Virgen del Rocio University Hospitals of Seville (code: TFG-ICT-2018-01).

### Data availability

The data associated with the article are not publicly available, although they are available from the corresponding author upon reasonable request.

## Results

**Figure 2:**
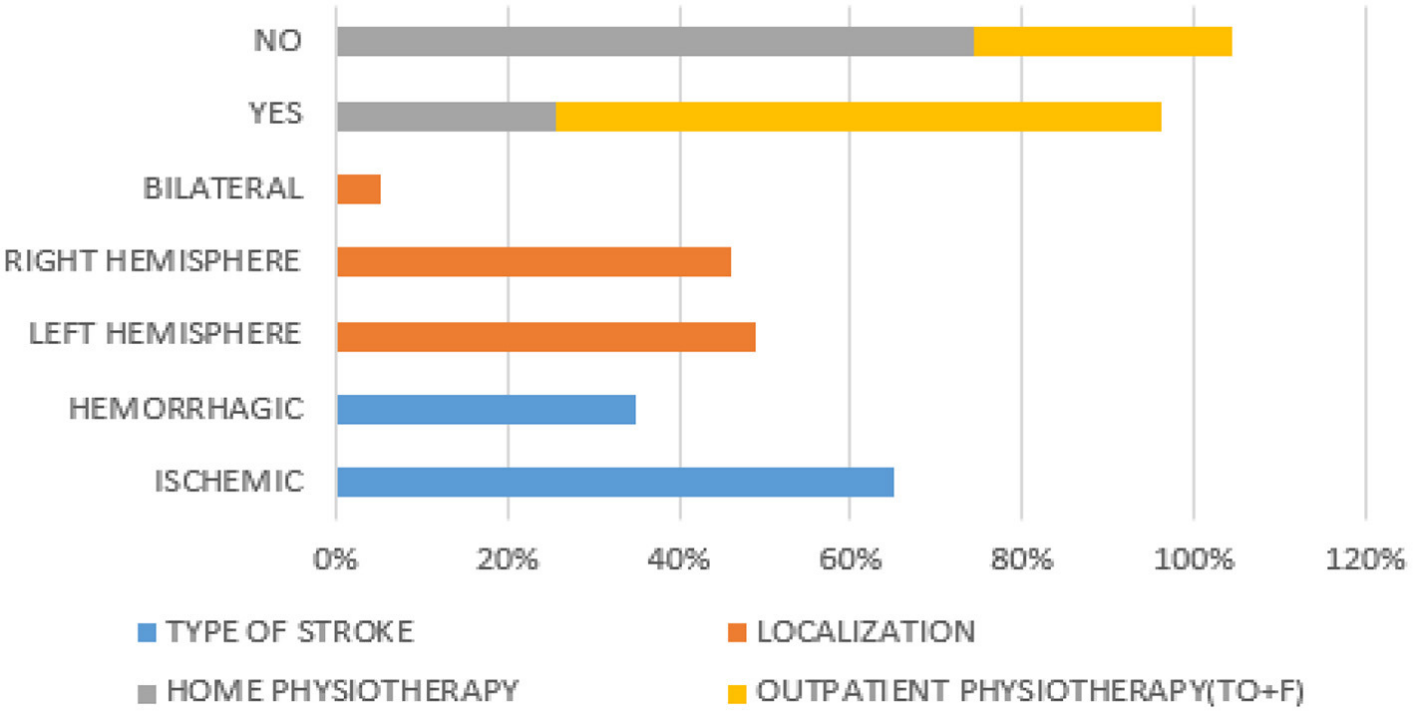
Proportional distribution of study outcomes.

One-hundred and thirty-seven pwS (79 men, 57.7%; 58 women, 42.3%; mean age 63.69 ± 12.377 years) were included in the study. The division of the sample is shown in Figure 2.

Of the 137 pwS, 107 received PT treatment during their hospital stay, with an average of 16.98 ± 23.063 treatment days. There was an average period of 9.91 ± 7.372 days between the stroke event and the beginning of the PT treatment. After the hospital discharge, the pwS waited 44.97 ± 27.087 days until the start of the outpatient PT treatment, which had an average duration of 121.27 ± 67.456 days (Table 1).

**Table 1:** Variables “stroke type” and “localization”: Student's *t*-test and weighted average.

	**TS**	**M ±SD**	**P_50_**	**Localization**	**M ±SD**	**P_50_**	**Median**	**SD**
Age	H	59.58 ± 12.06	59	B	61.71 ± 12.33	60	63.69	12.377
	I	65.69 ± 12.038	69	R	64.68 ± 10.65	66		
				L	62.96 ± 13.90	66		
Outpatient physiotherapy	H	134.40 ± 80.33	130.5	B	122.29 ± 36.45	147	121.27	67.456
	I	114.03 ± 58.433	106	R	11.56 ± 63.28	100		
				L	130.01 ± 72.88	122		
Hospital physiotherapy	H	26.02 ± 28.328	13.5	B	19.25 ± 12.31	15	16.98	23.063
	I	11.14 ± 16.689	6	R	14.46 ± 22.66	6.50		
				L	19.02 ± 24.04	10		
Early physiotherapy	H	12.93 ± 10.15	9	–	–	–	9.91	7.372
	I	8 ± 3.905	7					
Waiting time in the room	H	43.85 ± 26.715	39	–	–	–	44.97	27.087
	I	45.70 ± 27.515	39					

General statistical values of the study variables of the sample. TS, type of stroke; M ± SD, mean ± standard deviation; H, hemorrhagic; I, ischemic; B, bilateral; R, right; L, left.

The sample was differentiated based on the variables “stroke type” and “stroke localization,” performing a descriptive analysis and establishing correlations of each of them with the rest of the variables, especially with the duration (in days) of the different PT phases. With respect to the localization of the brain hemisphere affected, we differentiated among the right, left, and bilateral (massive) strokes. The mean number of PT sessions received, during both the hospitalization and outpatient phases, was larger in bilateral and left strokes (Table 1). A larger number of pwS had ischemic strokes with an affectation of the right hemisphere [45], followed by a left ischemic stroke [41] (Table 1).

The number of PT sessions received, both during the hospital stay and in the outpatient room, and the number of days between the stroke event and the start of the PT treatment (early PT) were larger in persons with hemorrhagic strokes. However, the waiting time between hospital discharge and the start of the outpatient PT was similar in both stroke types (Table 1).

The sample was differentiated based on the variables “stroke type” and “localization,” performing a descriptive analysis and establishing correlations of each of them with the rest of the variables, especially with the duration (in days) of the different phases of the PT treatment. A larger number of pwS had an ischemic stroke of the right hemisphere [45], followed by a left ischemic stroke [41] (Table 2).

**Table 2:** Contingency table: Localization type of stroke.

			**TS-H**	**TS-I**	**Total**
Localization	B	Count	4	3	7
		% Type of stroke	8.3%	3.4%	5.1%
	R	Count	18	45	63
		% Type of stroke	37.5%	50.6%	46.0%
	L	Count	26	41	67
		% Type of stroke	54.2%	46.1%	48.9%
Total		Count	48	89	137
		% Type of stroke	100.0%	100.0%	100.0%

TS, type of stroke; H, hemorrhagic; I, ischemic; B, bilateral; R, right; L, left.

To determine the correlations of “stroke type” with “age” and “outpatient PT,” Student's *t*-test was used (Table 3), obtaining a significant difference (*p* = 0.04) in patient age in relation to the type of stroke, with hemorrhagic strokes being less frequent (in younger pwS). Student's *t*-test was also applied to analyze the correlation between the variable “occupational therapy” and the number of sessions of outpatient PT performed, obtaining a significant result of *p* = 0.001 (Table 3). A total of 70.8% of patients received outpatient occupational therapy in combination with PT (Table 1).

**Table 3:** Correlation of the number of outpatient physiotherapy sessions performed for each stroke type with “age” and “occupational therapy” (Student's *t*-test).

	**TS**	***N***	**Median**	**Mean (SD)**	***P***
Age	H	48	59.58	12.063	**0.04** ^*^
	I	89	65.90	12.038	
Outpatient physiotherapy	H	48	134.40	80.330	0.127
	I	87	114.03	58.433	
Outpatient physiotherapy with OT	–	96	138.19	69.355	–
Outpatient physiotherapy without OT	–	39	79.64	38.974	**0.001** ^*^

TS, type of stroke; H, hemorrhagic; I, ischemic; N, number of cases; OT, occupational therapy; SD, standard deviation; P, p-value of Student's t-test. The bold values are statically significant values (< 0.05). The symbol ^*^ is for the bold statically significant values.

The correlation with the waiting days between the end of the hospital PT and the start of the outpatient PT was conducted with the Mann–Whitney *U*-test (Table 4).

**Table 4:** Correlation between the type of stroke and the different hospital physiotherapy variables (Mann–Whitney *U*-test).

	**Type of stroke**	***N***	**Average range**	***P***
Hospital physiotherapy	H	42	66.32	**0.01** ^*^
	I	65	46.04	
Early hospital physiotherapy	H	41	60.12	0.77
	I	65	49.32	
Waiting time in the room	H	41	51.68	0.824
	I	63	53.03	

H, hemorrhagic; I, ischemic; N, number of cases; P, Mann–Whitney U-test. The bold values are statically significant values (< 0.05). The symbol ^*^ is for the bold statically significant values.

There was a statistically significant difference between the number of hospital PT sessions received and the type of stroke, with a larger number of sessions being received by people with hemorrhagic stroke (*p* = 0.01).

The correlation of “stroke localization” with “age,” “outpatient PT,” and “hospital PT” was analyzed using the Kruskal–Wallis *H*-test, obtaining no statistically significant differences in these correlations. The duration of both PT phases was longer for bilateral (massive) and left strokes (Table 5).

**Table 5:** Correlations of “stroke localization” with “age,” “outpatient physiotherapy,” and “hospital physiotherapy” (Kruskal–Wallis test).

	**Localization**	***N***	**Average range**	***P***
Age	B	7	61.64	0.727
	R	63	71.62	
	I	67	67.31	
	Total	137		
Outpatient physiotherapy	B	7	74.00	0.299
	R	61	62.25	
	I	67	72.61	
	Total	135		
Hospital physiotherapy	B	4	75.63	0.133
	R	48	48.52	
	I	55	57.21	
	Total	107		

B, bilateral; R, right; L, left; N, number of cases; P, Kruskal–Wallis test.

The variable “age” was divided into two subgroups (pwS < 65 years of age and pwS ≥ 65 years of age), and it was correlated with the number of sessions of outpatient PT, the number of waiting days from the end of the hospital PT, and the start of the outpatient PT treatment. To this end, Spearman's correlation coefficient (rho) was applied, obtaining no statistically significant differences between these variables (Table 6).

**Table 6:** Correlation of “age” with the number of sessions of outpatient physiotherapy and the number of waiting days (Spearman's correlation coefficient).

**Age**	**Outpatient physiotherapy**	**Waiting time in the room**	***P***
< 65	132.67 ± 75.423	41.17 ± 24.438	0.064
≥65	110.04 ± 56.914	48.77 ± 29.24	0.778

P, Spearman's correlation coefficient.

## Discussion

At the descriptive level, the results are in agreement with those obtained in previous studies regarding the epidemiology and rate of stroke. The average age of the pwS (63.69 years), the greater prevalence of ischemic stroke (65%) over hemorrhagic stroke (35%), and the greater frequency in male patients (57.7%), except between the age group over 85 years, are in line with those reported in previous studies conducted in Spain (24, 25) and in nearby countries (26–28).

At the regional level, it is recommended to evaluate the pwS as soon as possible, preferably in the first 24–48 h (except in cases of severe complications), and prescribe the start of a PT treatment (29). In the hospital scope, the scientific literature (18, 30–35) highlights the physical and psychological benefits of early mobilization after suffering a CVA. Bernhardt et al. (35) analyzed 30 clinical practice guides, of which 22 recommended early PT (to be started in the first 48 h after the CVA), whereas the other eight advised very early PT (in the first 24 h after the CVA). There is no consensus on the optimum time to start the PT treatment after a stroke (36). The AVERT study (37) (A Very Early Rehabilitation Trial) shows negative results in pwS with severe affectation or with intracerebral hemorrhage subjected to early intensive mobilization, whereas Murie-Fernandez et al. (38) concluded that, for each day of delay in the start of the rehabilitation treatment, the functional prognosis of the person at discharge is worse. This corroborates the need to base this decision on the characteristics of each pwS and establish a patient profile that benefits especially from an early PT treatment. Thus, there is no consensus at the international level on the start of early PT after a stroke. Most of the clinical practice guides recommend beginning mobilization 24 h after the stroke event, as soon as the vital problems are under control (39), whereas important studies such as AVERT (40) do not associate very early mobilization with a significant reduction of post-stroke disability. In any case, the results of our study show an average of 9.91 days from the stroke event to the start of the hospital PT (“early PT”), which was slightly lower in the ischemic strokes (7 days); this could be related to the greater severity of the symptoms of the hemorrhagic cases and the consequent delay in the patient stabilization (41). These results are consistent with the waiting times mentioned in subsequent studies (42). Thus, we propose starting the PT treatment as early as possible, with a more direct referral to the PT service. There is a commitment on the part of such service to carry out the first PT assessment and treatment no later than 24–48 h after receiving the request.

The number of PT sessions conducted during the hospital stay was larger in the pwS affected by hemorrhagic stroke and in those who presented bilateral affectation (massive stroke). Since the hospital PT treatment is administered until the pwS is discharged, we presume that, as they are cases of greater clinical severity, the duration of the hospital stay is longer and, consequently, the duration of this phase of the PT treatment is also longer. Previous studies (43) have shown that the appearance of a greater number of medical complications in this type of person tends to prolong their hospital stay (43). Given the heterogeneity in the use of evaluations in CH, the functional state of the pwS upon discharge could not be reported in this study; therefore, we cannot conclude that a larger number of hospital PT sessions also entails a larger number of subsequent outpatient PT sessions for the recovery of the patient.

Regarding the duration of the PT treatment, there was also a slight increase in the number of PT sessions received in the room by the pwS affected by hemorrhagic strokes compared to ischemic strokes; this is in line with the results of the literature, which reports a better functional prognosis in the long term for hemorrhagic strokes (32) as the hemorrhage resolves, the brain compression decreases, and the neurological functions are recovered, which could justify the continuity of the PT treatment due to the slower but favorable evolution of the process. The recovery from ischemic strokes is, on the other hand, faster in terms of evolution in time (44), which is in line with the results of our study, showing that these pwS required a smaller number of outpatient PT sessions to recover.

Taking into account the differences with respect to the localization of the brain injury, the number of hospital and outpatient sessions performed with respect to the localization of the stroke was not significant. However, in both cases (hospital and outpatient PT), the average number of PT sessions conducted was slightly larger in the bilateral and left strokes. Previous studies have shown that left strokes present better functional results in terms of locomotion and posture recovery (45, 46), which could justify the larger number of PT sessions received and the decision to maintain them under treatment, given the good evolution of the process and the recovery potential.

The analyzed CH did not provide information about the PT techniques used in the subacute phase of the stroke, and the literature shows evidence of the effectiveness of task-oriented motor re-learning techniques over conventional PT (47) and the combined use of the new virtual and robotic therapies (48), which could shorten the treatment duration and improve the functional results.

Regardless of the stroke localization, there is evidence of an interdisciplinary program of PT and occupational therapy conducted in the hospital that produces better functional results (49). In our study, most of the persons in the outpatient phase also received an occupational therapy treatment at the same time, although in small groups and independent from the PT treatment.

In the organizational planning, the early inclusion of pwS under 65 years of age was already considered based on the hypothesis that, due to neuronal plasticity, the recovery capacity of the younger pwS would be greater if the PT treatment began earlier (13, 50). Our results do not show significant differences between the pwS < 65 years and the number of outpatient PT sessions performed, although the resulting values are close to significance. These pwS carried out a larger number of PT sessions and their waiting time was slightly shorter. Due to the insufficient information provided by the analyzed CH about scales of functional valuation upon discharge from outpatient PT, we cannot draw objective conclusions about the greater recovery capacity of younger pwS.

The heterogeneity in the functional evaluations carried out during the PT treatment of the analyzed CH was the key limitation of this study since it hindered the establishment of objective conclusions regarding the functionality of the pwS at the start and end of the different PT phases.

As a future research line, it would be necessary to homogenize the existing functional scales for pwS and apply them unavoidably both in the initial PT evaluation and at the end of the treatment.

We can conclude that the PPP for the early start of the outpatient PT in our service is that of a young pwS, with left or bilateral hemorrhagic stroke. A more direct referral to the PT service could shorten the waiting times. The establishment of a definitive profile requires the homogenous valuation of the functional state at the beginning and end of the PT treatment, thus establishing the PPP that would benefit more from an early start and a larger number of sessions, with the latter aspect being justified by the greater recovery potential of such PPP. Future studies should carry out homogeneous functional reviews that justify the efficient way to rehabilitate the PPP.

## Data availability statement

The raw data supporting the conclusions of this article will be made available by the authors, without undue reservation.

## Ethics statement

The studies involving human participants were reviewed and approved by Institutional Research Ethics Committee of Virgen Macarena and Virgen del Rocio University Hospitals, of Seville, Spain, (protocol code TFG-ICT-2018-01). Written informed consent for participation was not required for this study in accordance with the national legislation and the institutional requirements.

## Author contributions

MP-M, CL-M, IE-P, EM, DL-A, AG-M, and EP-P reviewed and edited the manuscript. AG-M funding acquisition. All authors approved the final version of the manuscript.
